# Transmission Selects for HIV-1 Strains of Intermediate Virulence: A Modelling Approach

**DOI:** 10.1371/journal.pcbi.1002185

**Published:** 2011-10-13

**Authors:** George Shirreff, Lorenzo Pellis, Oliver Laeyendecker, Christophe Fraser

**Affiliations:** 1 Medical Research Council Centre for Outbreak Analysis and Modelling, Department of Infectious Disease Epidemiology, Imperial College, London, United Kingdom; 2 National Institute of Allergy and Infectious Diseases, National Institutes of Health, Baltimore, Maryland, United States of America; 3 Department of Medicine, Johns Hopkins University School of Medicine, Baltimore, Maryland, United States of America; Eötvös Loránd University, Hungary

## Abstract

Recent data shows that HIV-1 is characterised by variation in viral virulence factors that is heritable between infections, which suggests that viral virulence can be naturally selected at the population level. A trade-off between transmissibility and duration of infection appears to favour viruses of intermediate virulence. We developed a mathematical model to simulate the dynamics of putative viral genotypes that differ in their virulence. As a proxy for virulence, we use set-point viral load (SPVL), which is the steady density of viral particles in blood during asymptomatic infection. Mutation, the dependency of survival and transmissibility on SPVL, and host effects were incorporated into the model. The model was fitted to data to estimate unknown parameters, and was found to fit existing data well. The maximum likelihood estimates of the parameters produced a model in which SPVL converged from any initial conditions to observed values within 100–150 years of first emergence of HIV-1. We estimated the 1) host effect and 2) the extent to which the viral virulence genotype mutates from one infection to the next, and found a trade-off between these two parameters in explaining the variation in SPVL. The model confirms that evolution of virulence towards intermediate levels is sufficiently rapid for it to have happened in the early stages of the HIV epidemic, and confirms that existing viral loads are nearly optimal given the assumed constraints on evolution. The model provides a useful framework under which to examine the future evolution of HIV-1 virulence.

## Author Summary

Recent studies have suggested that virulence in HIV-1 is partly a characteristic of the virus which is carried from one infection to the next. An infection with intermediate virulence will produce more transmissions during the infectious lifetime because it optimises the trade-off between rate of transmission and duration of infection. Natural selection acts on the heritable variation to increase the relative prevalence of strains with intermediate virulence. In this study we model the evolution of virulence in the viral population as these more successful strains are preferentially transmitted. We fit this model to data from transmitting couples, and find that the model fits the data well. We use this fit to estimate the contribution of the host and the virus to virulence, which complements recent estimates of the heritability of virulence. We also estimate the rate at which the viral determinants of virulence evolve between infections, and this provides predictions for how rapidly the virulence of HIV-1 evolves in a population. We suggest that natural selection on transmissibility results in substantial evolution of virulence in the population. This is sufficiently rapid for virulence to have reached current levels over the available timescale of the human epidemic.

## Introduction

The median time between HIV-1 seroconversion and progression to symptomatic Acquired Immune Deficiency Syndrome (AIDS) is approximately 10 years [1]. However, there is considerable variation in this rate of progression, with substantial proportions of infected individuals progressing to AIDS in less than 5 years, or remaining AIDS-free after 20 years. Explaining this variability is an important goal of HIV pathogenesis research. Many cofactors which influence time to AIDS have been identified e.g. host genetics [2], host age [1], and recently viral factors have been implicated [3]–[10].

In this paper we explore the extent to which viral factors which influence virulence, changing from one infected individual to the next, may have evolved under natural selection in the early phase of HIV-1's history. Between-host selection, leading to changes in the virulence of HIV-1, has potential major implications for the number of human life years affected.

Virulence is often defined as the excess mortality of the host which occurs as a result of infection with a pathogen. In the case of HIV the excess mortality is nearly 100%, so virulence can be better defined by the reciprocal of the time from infection to death, or time to AIDS. However, since this can only be defined at the host's death, we use set-point viral load (SPVL) as a proxy for virulence. This refers to the relatively stable density of virions in the blood which characterises asymptomatic infection. There is considerable population level variation in SPVL, in spite of its relative stability within the individual [11]. SPVL is widely used as a prognostic indicator for AIDS, as individuals with a higher SPVL have a higher rate of CD4+ cell decline, and they tend to progress more rapidly to AIDS [12], [13] and die sooner as a consequence [14]. As a result of its relative constancy during asymptomatic infection, SPVL can be measured at a wide range of time points in an individual's infection [15].

A simple conceptual model of how SPVL may evolve by between-host natural selection (i.e. selection for the more transmissible genotypes) requires consideration of the transmission potential of individuals of different SPVL. The transmission potential, defined as the product of duration of infection and infection rate, increases with either component of this product. A positive correlation between SPVL and transmission rate has been convincingly demonstrated within heterosexual couples with initially discordant serostatus [16]–[18]. Since there is also a negative correlation between SPVL and duration of asymptomatic infection [12], there is therefore a trade-off between duration of and transmission rate during asymptomatic infection. Previous work has quantified this trade-off to suggest that SPVL most commonly observed in infections maximise the transmission potential, suggesting that the distribution of SPVL was shaped by natural selection [19].

Natural selection requires that a trait has heritability from one generation to the next, in addition to variation and differential reproductive success. A number of recent studies have identified and quantified this heritable component of SPVL variation which is maintained from one infection to the next [3], [5], [6], [9], [10].

Recent studies from the Netherlands [20] and Italy [21] have found that the mean log_10_ SPVL has increased over the recorded history of an HIV-infected cohort, and the rate of CD4+ cell decline has increased. However different transmission groups have demonstrated different patterns of evolution of SPVL. In the initial stages of the epidemic (mid 1980s) injecting drug users showed slower CD4+ declines than heterosexuals or men having sex with men, but this difference decreased over the subsequent decade [21]. A study with similar methodology in Switzerland found stable virulence over the same time period [22]. This suggests that such trends may be area- and risk-group specific. In two studies showing an increase, the levels of SPVL in the earlier time points are lower [20], [21] than those which are optimal for transmission [19]. Various studies of the rate of CD4+ decline also suggest an increasing virulence [23], [24]. A study of the *in vitro* replicative fitness of viruses sampled at different time points reported a decrease in replicative fitness over the course of the epidemic in Amsterdam [25] although a subsequent study of the same city which controlled for time of seroconversion found an increase [26]. Overall, observational results on changing virulence are inconclusive, though they suggest either an equilibrium or a slow increase in that direction.

The lack of evidence for consistent population level trends in SPVL evolution [21], [22] suggests a) the global distribution of SPVL has stabilised at an equilibrium level; b) the rate of evolution is very slow or c) the distribution of SPVL is determined by factors which do not evolve. However, we think c) unlikely, first due to the observations on the heritability of SPVL described above, and second because there is evidence for evolution of SPVL occurring in particular areas or risk groups [20], [21].

To address the expected dynamics of SPVL evolution, we developed and analysed a deterministic mathematical model of between-host transmission and evolution incorporating known parameters linking SPVL to the duration of infection and the rate of transmission. The broad aim was to investigate the hypothesis that viral genotypes of intermediate virulence are naturally selected by transmission [19].

The primary of aim of this study was to use the observed distribution of SPVL to estimate the quantities of unknown host and viral factors which affect the process of between-host evolution. Comparing the model to data allowed us to calculate the likelihood of the unknown parameters.

The secondary aim was to assess whether the model, under these parameter estimates, allows convergence of the SPVL distribution towards an intermediate level, or at least to slowly changing levels consistent with observational studies, regardless of the virulence of the founding strain, and whether this can occur within a plausible timescale. The estimated time of origin of HIV-1 is before the most recent common ancestor, which has been dated to 1908 with 95% confidence interval 1884–1924 [27]. If evolution has occurred between the founding strain and current infections then it has occurred over a period of ∼100 years.

## Results

We modelled the dynamics of putative genotypes of HIV-1 which differ from one another in their mean log_10_ SPVL. SPVL was assumed to vary as a result of both host and virus factors. These genotypes differ in their reproductive success as a result of the dependency of duration of asymptomatic infection and transmission rate on SPVL. Their prevalences change over time through competition for susceptible individuals in a constant population.

The model is formulated as a standard HIV epidemic model in which different viral strains or genotypes compete. Virulence is considered as a one-dimensional trait, with each genotype represented by a point on the one-dimensional spectrum of increasing virulence. When a person is infected by a virus of a given genotype, the infection is characterised by a SPVL which reflects the virulence, but also other non-viral factors. When transmitted, the virus can also mutate to higher or lower levels of virulence.

The model encodes the natural history of infection. After infection, individuals experience a brief period of highly infectious acute stage, after which they progress to chronic asymptomatic infection. Their SPVL determines both the duration and infectiousness of this asymptomatic stage, after which their viral load and infectiousness increases again as they progress to AIDS and death. Individuals are assumed to engage in serially monogamous partnerships; a realistic description of the sexual network was not an aim of this study.

For the sake of parsimony, we focused on relatively simple mathematical models with minimal sets of parameters, and thus left some important questions open for further study. In particular, we did not explore the effect of population structure, stochastic fluctuations, differences between subtypes, superinfection, and founder effects, and we considered only the situation of natural, untreated infection, thus appropriate to describing the evolution of the virus prior to the widespread adoption of antiretroviral therapy. We also did not address the question of conflicting directions of selection at the within and between host level, describing in-host changes in virulence instead as random drift. We hope to address these important questions in future work.

### Variance decomposition

A useful practical and conceptual approach to interpreting various influences acting on SPVL is to decompose the total observed variance, *σ_P_* ^2^, into its components, genotypic, mutational and environmental variance (*σ_G_* ^2^, *σ_M_* ^2^, and *σ_E_* ^2^) [28].
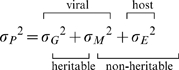
(1.1)Genotypic variance *σ_G_* ^2^ refers to differences in SPVL between infected individuals caused by viral factors which are preserved from one infection to the next. Environmental variance, *σ_E_* ^2^, refers to any source of SPVL variance external to the virus. Host factors e.g. age [29], sex [30] and host genotype [31], in particular HLA type [2] contribute significantly to variation in SPVL between individuals, and there may be other human and non-human covariates of SPVL e.g. antigenic stimulation [32]. All of these factors, extrinsic to the virus, contribute to *σ_E_* ^2^ in our terminology.

Mutational variance, *σ_M_* ^2^, accounts for changes in the viral virulence genotype which result from mutation of the virus between one generation and the next (i.e. one infected host and the next) as a result of within-host replication and selection of the virus. Since the viral determinants of SPVL are not currently known, this cannot be related to the nucleotide substitution rate.The mutational standard deviation, σ_M_, is simply the expected difference in the viral component of SPVL between an index and a secondary infection.

Heritability, *h* ^2^, which has been quantified in previous studies, was defined as the fraction of variance explained by shared viral factors within a transmitting couple [6], [33]. We estimate *h^2^* as the proportion of variance in SPVL explained by heritable viral genetic factors:
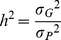
(1.2)Alternative definitions of heritability, including the proportion of variance in SPVL explained by the SPVL of the index case, and the proportion explained by viral factors, are discussed and estimated in Text S1.

In this study, we aim to separately estimate *σ_M_* ^2^ and *σ_E_* ^2^, and thus gain a better estimate of the extent of viral factors in individual infections, and the parameters needed to predict evolution.

### Model fitting and parameter estimation

The primary aim of the analysis was to quantify the effects of host and virus on variation in SPVL. The values of the environmental and mutational standard deviations (*σ_E_* and *σ_M_*) were estimated using a maximum likelihood approach. Since the model predicts not just the distribution of SPVL, but how they change from one infection to the next, the model could predict the observed SPVL in both index and recipient partners in transmitting couples.

**Figure 1:**
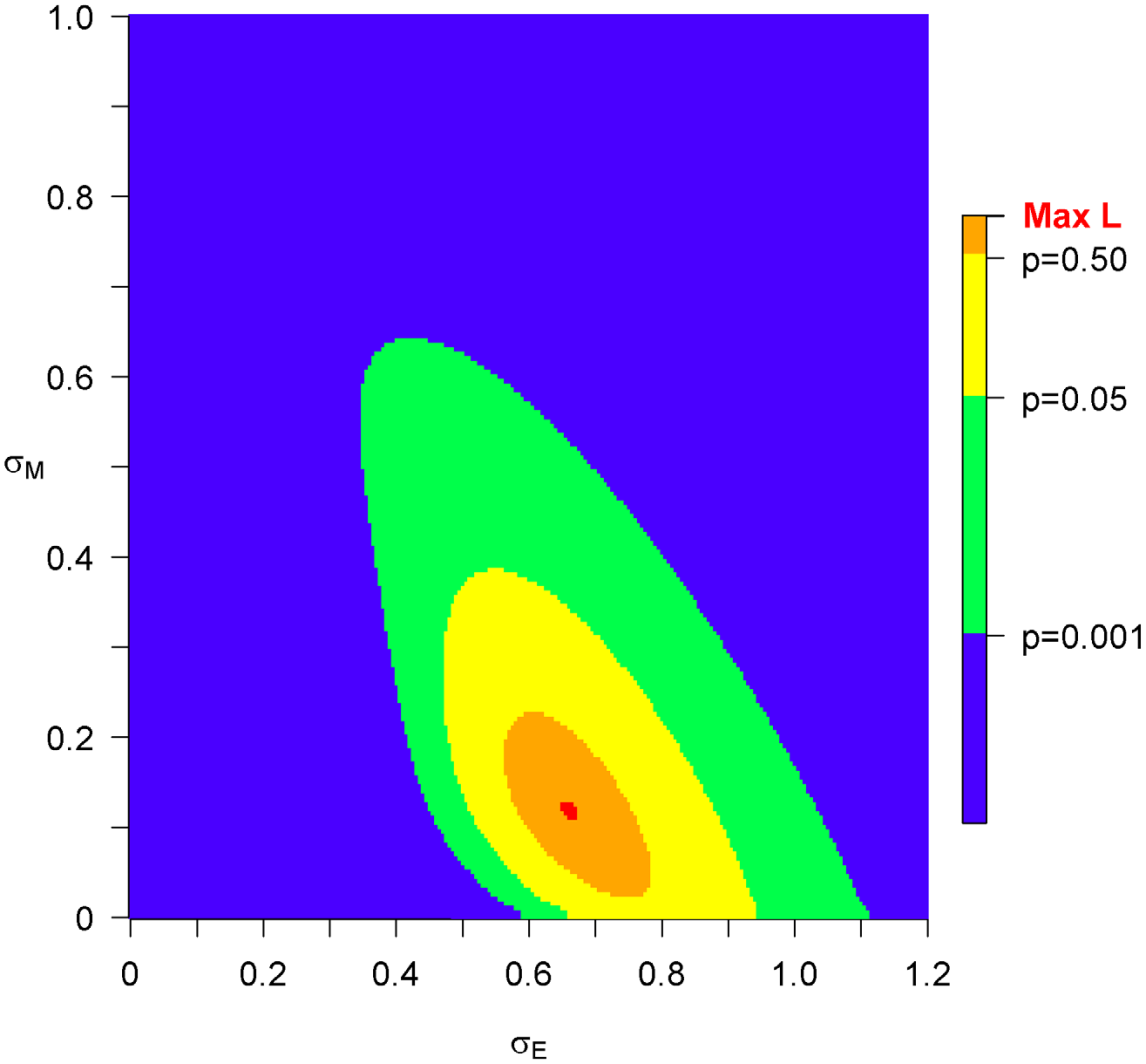
Likelihood surface for the environmental and mutational standard deviation (*σ_E_* and *σ_M_*). The maximum likelihood estimate is represented by the red point, and the regions of 50%, 95% and 99.9% confidence in orange, yellow and green respectively. The method for calculating confidence intervals is given in **Text S1** equation (5.4).

**Figure 1** shows the likelihood surface for the environmental and mutational standard deviations (*σ_E_* and *σ_M_*), and the bivariate confidence bounds. The maximum likelihood estimates are *σ_M_* = 0.12 (95% confidence interval 0.00 to 0.39) and *σ_E_* = 0.66 (95% confidence interval 0.47–0.94). The estimates with highest mutational standard deviation within the 95% confidence bounds are *σ_M_* = 0.39 and *σ_E_* = 0.55 referred to later as the most mutable plausible scenario. Further details of the likelihood surface are given in **Figure S2**. The diagonal nature of the region of high likelihood in **Figure 1** (or better viewed in **Figure S2**) indicates a trade-off between the two parameters in terms of the quality of model fit.

**Figure 2:**
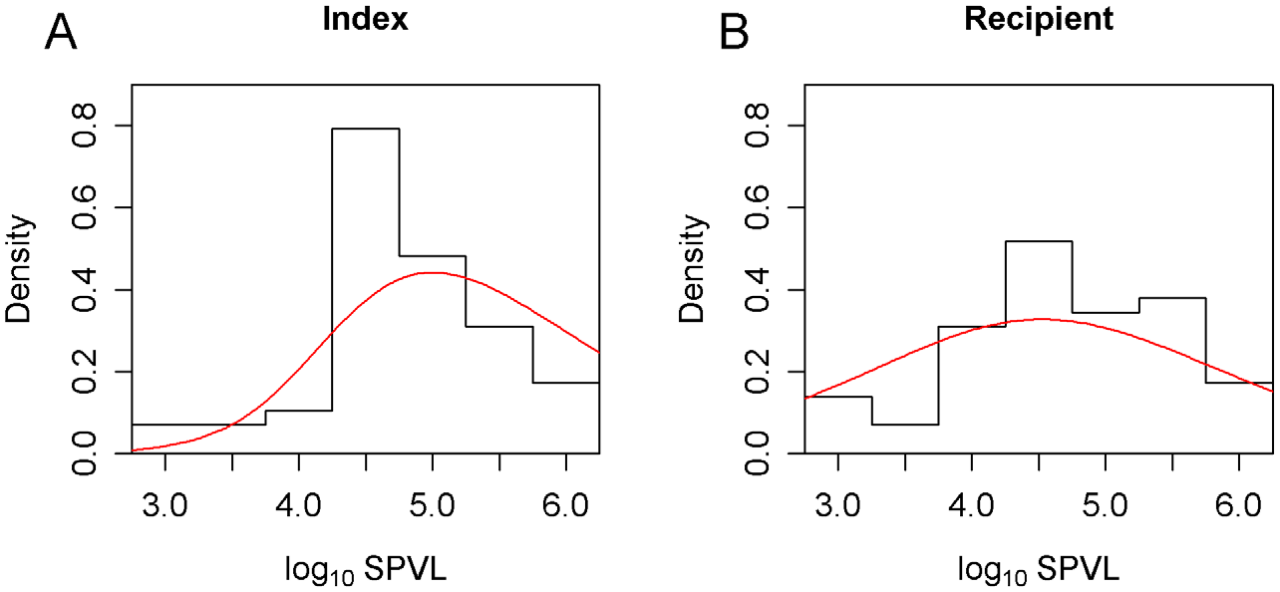
Fit of the model (red line) to data (black line). (**a**) The distribution of SPVL in the index partner, and (**b**) the recipient. Where these roles are unknown, each individual in the pair represents half an individual in each figure. The modelled distributions were calculated from equations (3.5) and Text S1 (5.5) for the recipient and index partner, respectively.

**Figure 2** shows the quality of fit of the model to the distribution of SPVL in index partners and recipients in transmitting couples, and the estimated heritability was 26% (compared to 27% in a previous statistical analysis of these couples [6]). We conclude that the model describes the data well. The distribution and heritability of set-point viral load is well described by a multi-strain model of HIV-1 virulence evolution.

### Convergence of SPVL distribution

Having derived maximum likelihood estimates of parameters from an equilibrium solution to the model, the dynamics of genotype competition were then simulated numerically in order to assess whether or not convergence would occur under those parameter values, and on what timescale the convergence would occur.

**Figure 3:**
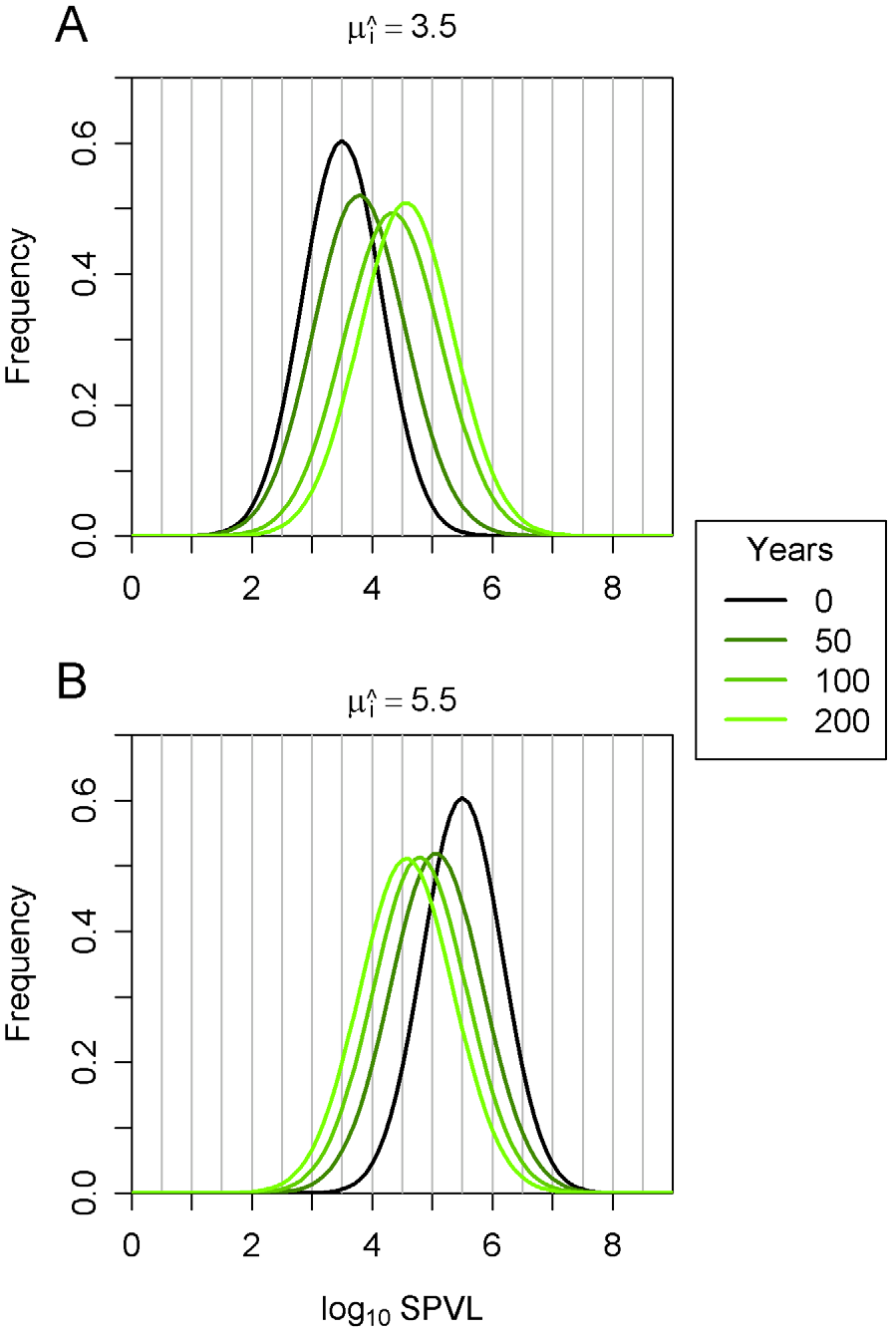
The evolving distribution of SPVL. The SPVL distribution evolves in the population over the years since introduction of the founding genotype. Maximum likelihood values from **Figure 1** were used (*σ_M_* = 0.12, *σ_E_* = 0.66). The mean log_10_ SPVL of the founding genotype was (**a**) 3.5 and (**b**) 5.5.

The evolution of the SPVL distribution is shown in **Figure 3**. Regardless of whether the virulence of the founding genotype was high or low, the SPVL evolved towards an intermediate level with a mean log_10_ SPVL of 4.5.

**Figure 4:**
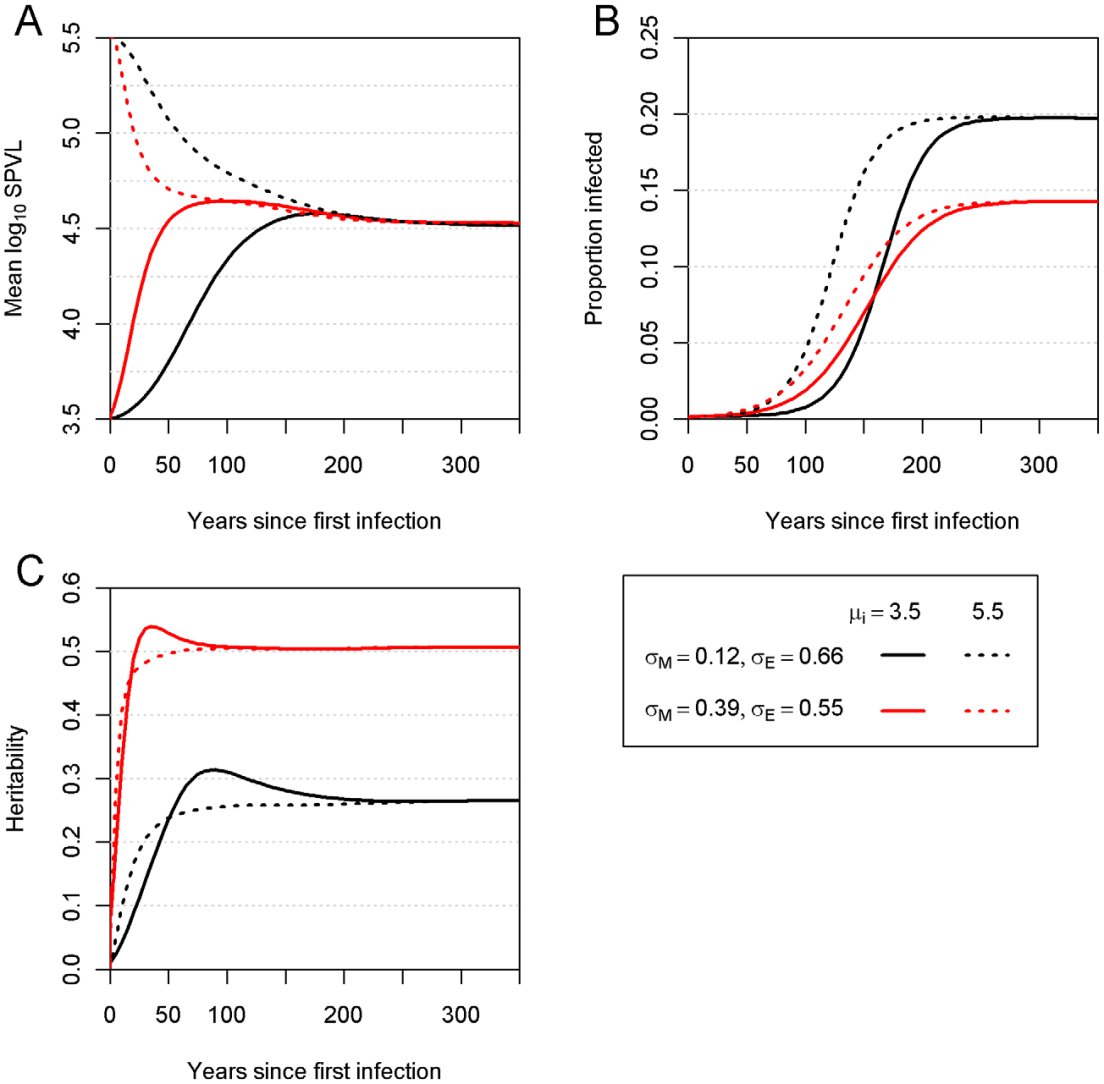
Mean log_10_ SPVL and heritability over time. (**a**) Mean log_10_ SPVL, (**b**) heritability. The epidemic was run under maximum likelihood parameter (*σ_M_* = 0.12 and *σ_E_* = 0.66, black), or the combination of parameters with maximum *σ_M_*, consistent with high likelihood (*σ_M_* = 0.39 and *σ_E_* = 0.55, red). The solid lines show runs in which the founding genotype had *μ* = 5.5, while the dashed lines show runs with a founding genotype with *μ* = 3.5.

This convergence on intermediate SPVL values also occurred when other combinations of parameter values in the region of high likelihood (**Figure 1**) were used instead. The rate of convergence was positively related to *σ_M_*, as shown in **Figure 4(a)**, where the maximum likelihood prediction is compared to the most mutable plausible scenario. Convergence towards intermediate virulence occurred in approximately 150 years under the maximum likelihood values. There was still change in the mean after this time but runs beginning with high or low virulence converge around this time point. The same point was reached in 50 years under the most mutable plausible scenario.

The heritability was also calculated over time (**Figure 4(b)**) and under maximum likelihood values of *σ_E_* and *σ_M_* this reached equilibrium at 26%, which is consistent with previous studies [3], [5], [6], [9], [10]. Further details of the heritability and variance at equilibrium are given in **Figures S3 and S4**.

**Figure 5:**
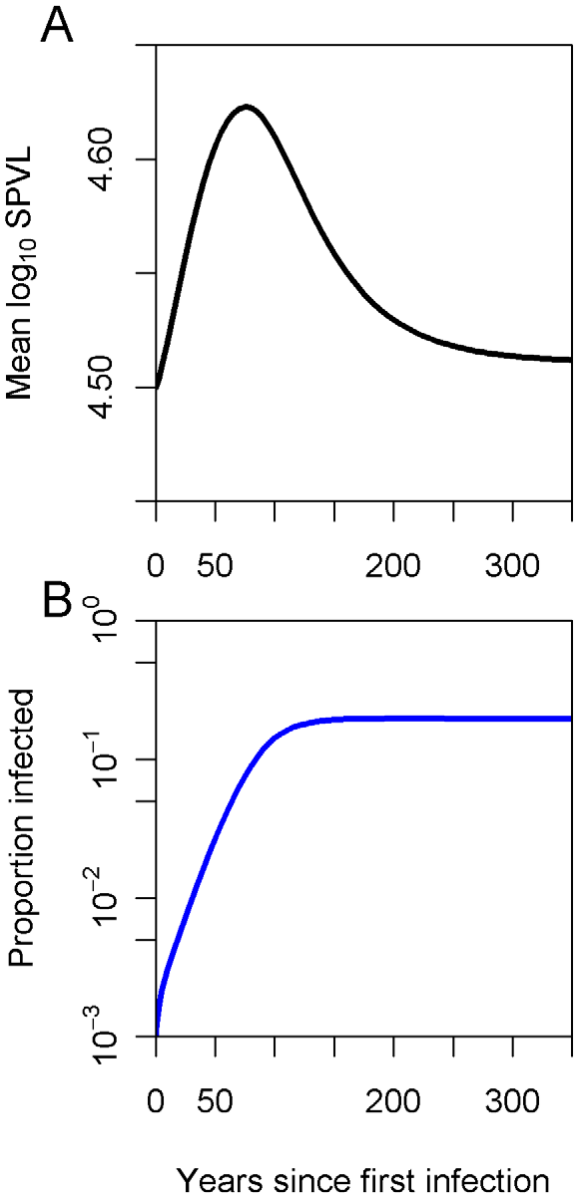
The evolution of the mean and the epidemic growth pattern. The founding genotype has *μ* = 4.5, very close to the equilibrium mean, to illustrate changes in the mean in response to growth or shrinkage of the epidemic. (**a**) The evolution of the mean log_10_ SPVL during epidemic growth (**b**).

In order to examine how changes in mean log_10_ SPVL are related to the stage of the epidemic, we examined the effect of proportion infected over time. The effect was most evident when the founding virulence closely matched the equilibrium virulence (**Figure 5(b)**). During the epidemic growth phase the mean virulence increased to levels above the optimum, and then returned to the optimum as the proportion infected reached equilibrium.

**Figure 6:**
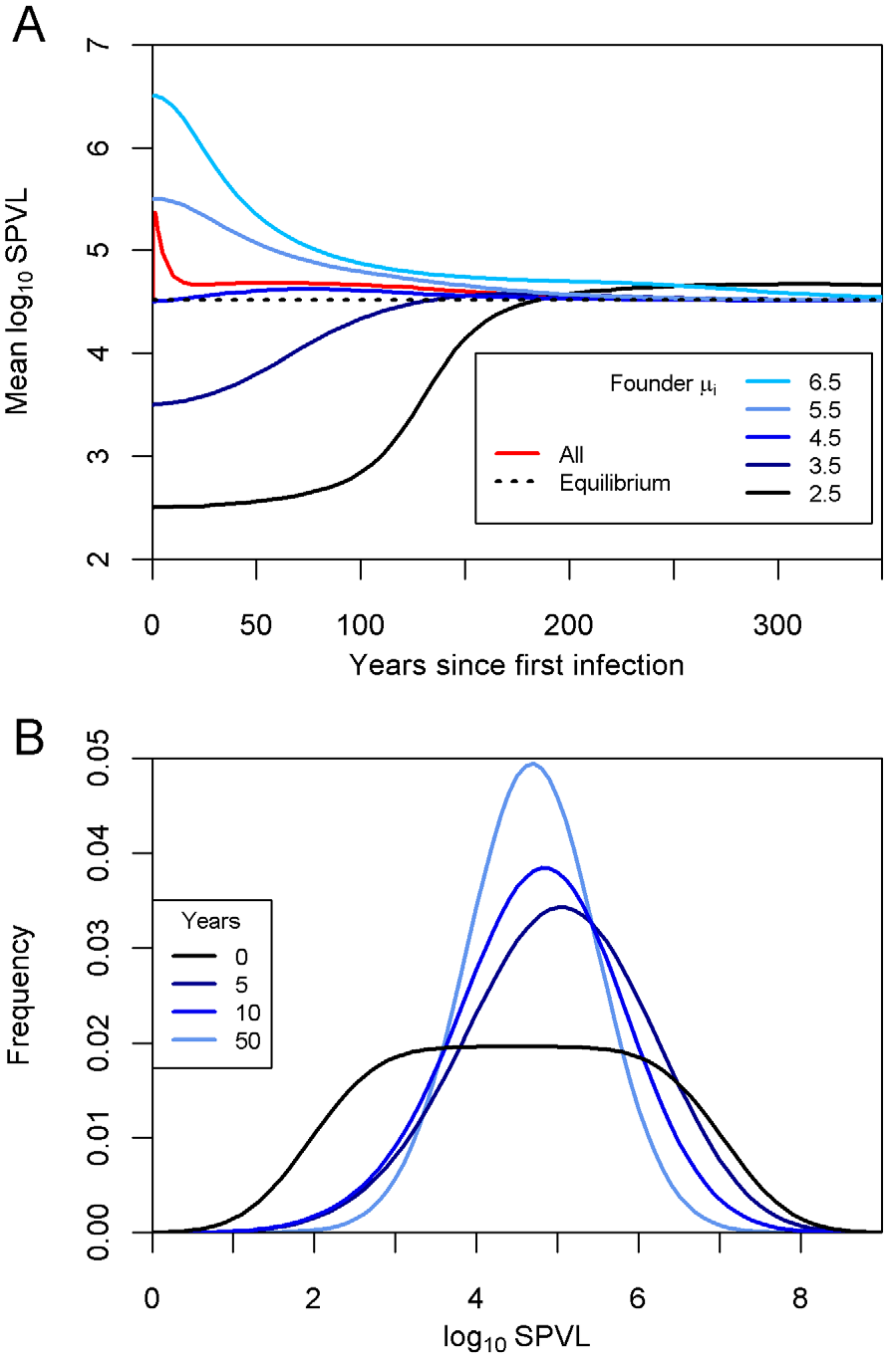
Evolution of SPVL from various scenarios of founding genotype. (**a**) Mean log_10_ SPVL over time for different founding virulences. These range from *μ* = 2.5 to 6.5 log_10_ SPVL, “All” (red) begins with all genotypes from *μ_i_* = 2.0 to 7.0 at equal prevalence, and “Equilibrium” (dashed black) is the SPVL value to which all scenarios in this figure are evolving. (**b**) Evolution of SPVL distribution from high diversity scenario where all genotypes are equally represented at the start, corresponding to “All” in panel (**a**). The parameter values for both are maximum likelihood values, *σ_M_* = 0.12 and *σ_E_* = 0.66.

We varied the founding virulence to investigate its effect on rate of convergence (**Figure 6(a)**). This had a marked effect on how quickly the mean log_10_ SPVL reached equilibrium (4.52 log_10_ SPVL). When the founding genotype had mean 4.5 log_10_ SPVL, equilibrium with regard to the mean was reached very quickly, and the more different the SPVL of the founding genotype, the longer the time to convergence. A similarly rapid convergence is seen if all genotypes had equal prevalence at the start of the run. The mean underwent little change (data not shown) but the variance rapidly decreased as the most successful genotype, already present in the population, began to dominate (**Figure 6(b)**).

Finally, we investigated the sensitivity of our findings to the choice of parameter values determining the dependencies of infectiousness and duration of asymptomatic infection on SPVL. These parameters were previously estimated from datasets from Amsterdam and Zambia [19]. Here, we tested the sensitivity to those estimates by bootstrapping these datasets, refitting the parameters each time and calculating the corresponding maximum likelihood estimates of *σ_E_* and *σ_M_*. Details of the method are in **Text S1** and **Table S2**. The resulting maximum likelihood estimates (**Table S3** and **Table S4**) are similar to those from the principal analysis (**Figure 1**).

## Discussion

In this paper, we developed a multi-strain evolutionary epidemiological model of HIV-1 virulence, and showed that it could accurately reproduce observations on the distribution of viral load and its heritability in transmitting couples (**Figure 2**). We were able to estimate the proportion of variance in set-point viral load explained by viral genetic factors (26%, 1−(*σ_E_* ^2^+*σ_M_* ^2^)/*σ_P_* ^2^), and separately how much these factors change (‘mutate’) from one infection to the next. Our best estimate is that virulence changes slowly towards an evolutionary optimum over decades, but we cannot rule out faster changes (**Figure 4** and **Figure 6**).

Our aim here was to develop a simple, parsimonious ‘broad-brush’ model to understand the principles of HIV-1 virulence evolution in a generalised epidemic using data currently available. Most of the parameters were derived from Sub-Saharan African studies (**Table S1**), suggesting that the model has most direct relevance for this context. This is our intention, as this is where most of the adaptation of HIV-1 to the human population has occurred. The parameters determining the curve of survival from disease progression were derived from European data, and since these data predate antiretroviral therapy they are not expected to differ substantially from parameters derived from Sub-Saharan Africa.

We do not expect the epidemic in other contexts to differ drastically. Two studies which have observed a change in virulence in the Netherlands [20] and Italy [21] appear to support our hypothesis as the virulence in both situations has risen from a sub-optimal level towards equilibrium, as predicted in our model. The same trend was not seen in Switzerland [22], however, and further work is required to apply the model rigorously to the European context with a view to explaining these trends. More realistic predictions will require more detailed models, and by necessity more data. We list some factors that could be included in a more detailed analysis.

Describing the differences between subtypes of HIV-1 seems like one of the biggest challenges to the model presented here. We considered virulence evolution on a single dimension of low-to-high, with single functions describing the relationship between viral load, infectiousness and duration of asymptomatic infection. HIV-1 subtypes in fact differ in their transmission parameters independently of their differences in SPVL [4], [7], [8]. Subtype A shows a slower disease progression when compared to other subtypes [34]. More specifically, data from the Rakai study showed that subtype A infection results in slower disease progression than subtype D even though the distribution of SPVL is the same [4], [7]. From the same cohort it was shown that subtype A is also more transmissible than subtype D even when viral load and other confounding variables are controlled for in a regression [35]. Subtype A is therefore fitter than D in both duration and transmissibility, and the evolutionary hypothesis would predict the gradual replacement of subtype D by subtype A, which has been observed in Uganda [36] and Greece [37]. Other noteworthy trends include the dominance of subtype C in southern Africa [38], which may be a result of an extended period of high viraemia in primary infection [39]. Taken together, these findings strongly suggest that HIV-1 virulence can change in ways not fully reflected by set-point viral load, and thus that more data are needed to identify other appropriate surrogate measures (or determinants) of virulence. More generally, the theoretical challenge is then to explain in terms of these other determinants of infectiousness and survival, how differences in virulence are maintained in different viral subtypes.

There are a number of other directions in which our model could be developed. In this study the mutational variance, the extent to which the viral genotype changed from one infection to the next, was considered independent of the age of infection (AOI). At first, this may seem a paradoxical choice, since mutation which occurs between hosts must be the result of mutations and selection occurring within the infected host. It would reasonable to suggest that the size of between-host mutation is positively related to the AOI, since nucleotide divergence from the founding strain has been shown to occur at a constant rate during infection [40]. If this were the case, the between-host mutation rate would be the same regardless of the generation time and consequently of the virulence of the virus. However, a study of within-host evolution over time found that the rate of divergence from the founding genotype was positively correlated with viral load [41], suggesting that higher virulence infections diverge more rapidly. A model with a mutational variance independent of the AOI allows for this, as a higher virulence virus will have more generations in a given amount of time and therefore more between-host mutation events.

An accurate functional representation of mutational variance as a function of AOI thus requires more detailed understanding than seems currently possible. To resolve this, and for the sake of parsimony, we assume that the two effects described above cancel each other out, and thus that the mutational variance is independent of AOI. To test the sensitivity to this assumption, we changed the model to include AOI-dependent mutational variance (linearly increasing as a function of time), and the results were qualitatively and quantitatively similar (data not shown).

An additional problem with this model is that the data to which the model is fitted consists of transmission pairs, for most of whom the age of infection at which transmission occurs is unknown. Assuming an AOI-independent mutational variance considerably reduces the complexity of the analysis. There is however little doubt that extending the model to include a more detailed description of within-host processes and also resolving the effects of conflicting selection at the within and between host levels will be enlightening.

The pattern of mutation was modelled as a log-normal distribution. It may be reasonable to assume that the distribution is negatively skewed because deleterious mutations are much more frequent than beneficial ones, for example in the case of protease gene [42]. However, it is misleading to compare the between-host mutation process to the mutation of individual viral genomes because deleterious mutations may be counterbalanced by within-host selection for viable viruses and there is no evidence for asymmetry in the net effect.

The host effect in this study was also modelled by a log-normal distribution which is justified if there are a large number of host effects and they are assumed to each have a multiplicative effect on SPVL. Host effects are known to account for a certain quantity of SPVL variation [2], [29]–[31], [43] and a very low estimate of the environmental variance would not be consistent with these studies. The maximum likelihood estimate of *σ_E_* was encouragingly high (*σ_E_* = 0.66, **Figure 1**), contributing 71% of the total variance in SPVL. As more is understood about how the host contributes to variation in SPVL, this source of variance may be further decomposed [31].

The epidemiological component of this model could be made more realistic. The model could for example be structured by age, sex, location, sexual activity, HLA type and include stochastic effects. It is not clear to us what effect on virulence these heterogeneities will have, but they might help for example explain the persistence of diversity between subtypes and help provide reasonable initial conditions, since a stochastic model could elucidate which viruses are more likely to have started the epidemic. The analysis could be further developed by relaxing the assumption that the SPVL is at an evolutionary optimal equilibrium, though we note that this assumption provides good agreement with data (**Figure 2**). We note that the mean log_10_ SPVL and its heritability do not change substantially in the later stages of the epidemic (**Figure 4a–c**), and the mean log_10_ SPVL of the Ugandan data (4.51) is close to the predicted equilibrium value (4.52), suggesting that even if the observed data do not represent an equilibrium, they represent something close enough to render the maximum likelihood parameter estimations reasonable.

Despite being simple and parsimonious rather than detailed, our model provides a general framework that makes use of the most recent data on the heritability of set-point viral load, and that can be used to interpret past and predict future trends in SPVL.

One interesting trend is that the mean log_10_ SPVL can be observed to increase above the equilibrium value for a short while during the early stages of the epidemic. Epidemic growth is expected to favour a higher virulence than at equilibrium as a result of the cumulative advantage of rapid transmission when hosts are abundant [19], [44]. This is better demonstrated in **Figure 5(c)** which shows the evolution of the mean log_10_ SPVL from a founding virulence very close to the equilibrium mean. At this level of resolution the temporary spike in virulence can be seen, and this corresponds to the period of epidemic growth. As the number of susceptible individuals grows and the epidemic begins to slow, the virulence decreases in response towards equilibrium as longer-lived genotypes are favoured.

This suggests that if SPVL can evolve at the between-host level then a growing epidemic could select for higher virulence viruses. Bolker et al. [44] model this phenomenon and suggest that the peak of this transient virulence is likely to occur late within the first exponential growth phase of the epidemic, so if this were observable the virulence is likely still to be in this transient state above the equilibrium. Whether this phenomenon has contributed to the recent increase in virulence in Italy and the Netherlands [20], [21] cannot be distinguished from an increase in virulence as a result of the founder having sub-optimal virulence. A future slight decrease in virulence as an epidemic saturates would provide evidence for this hypothesis, if it could be identified [44]. The optimum virulence could also be shifted by a widespread intervention which affects the nature of transmission such as circumcision, vaccination, or antiretroviral therapy. In the current study we introduced a model which may be used to predict such effects on virulence.

Recently published studies reporting the development of a reasonably effective vaccine [45] and a protective vaginal gel [46] are promising in the fight against HIV transmission. Hypothetically, a vaccine may offer more protection against lower virulence genotypes and select for more virulent ones, or vice versa. Gandon et al. [47] produced simple models which suggested that vaccines which target infection or transmission should have a negligible or negative effect on virulence as reducing the rate of transmission benefits pathogens which keep their host alive longer. However they also modelled vaccines which reduce the growth or the toxicity of the pathogen and suggest that this would select for pathogens which have higher virulence which would have a negative effect when unvaccinated individuals were infected.

Antiretroviral therapy during asymptomatic infection reduces transmission rate [48], [49], presumably by reducing viral load [50], [51]. Antiretroviral therapy would therefore modify the relationship between SPVL, transmission and duration of asymptomatic infection, and it is possible to construct hypothetical scenarios that could select for either increased or decreased SPVL. In summary, our model could be used to predict (in general terms) the effects different interventions would have on virulence. These changes are expected to be relatively modest compared to gains obtained by curtailing transmission, but nonetheless some consideration should be given to the possibility of increased virulence and whether it could be mitigated.

### Conclusion

Our results support the hypothesis that the distribution of SPVL, and by implication of HIV-1 virulence, can plausibly be explained by selection for increased transmission in populations, though differences between viral subtypes needs to be elucidated in future work. Our method disaggregates the effects of viral factors acting to determine SPVL, the effect of mutation (and thus indirectly within-host evolution), and other environmental and host factors. The best estimates indicate a relatively high proportion of SPVL explained by viral factors (26%), as well as a modest rate of evolution of putative viral virulence factors. Reconciling these findings with data on within-host viral evolution may yet shed further light on the role of viral factors in HIV-1 pathogenesis.

## Materials and Methods

### Viral genotypes and SPVL phenotypes

In order to simplify simulations, we modelled a discrete finite set of viral strains (‘genotype’), each capable of producing a finite range of possible SPVL (‘phenotype’).

Each infected host in the model carries a viral genotype, *i*, and has a phenotype, *j*. Hosts were not explicitly described in the model, rather the model specified the dynamics of relative prevalences of hosts infected with a virus of genotype *i* and phenotype *j*. In other words, we used a compartmental multi-strain epidemic model.

Each genotype is defined by a predisposition to give rise to higher or lower SPVL. Following the decomposition given by equation (1.1), viral loads can be given as:
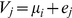
(2.1)where e_j_ is the environmental component (with mean zero and variance *σ_E_* ^2^) and *μ_i_* is the component attributed to viral factors. For a population of individuals infected with viral genotype *i*, the mean log_10_ SPVL will be given by *μ_i_*, which is therefore a natural measure of the virulence of genotype *i*. For two viral genotypes *i* and *k* such that *i* is more virulent than *k*, i.e. *μ_i_*>*μ_k_*, not all individuals infected with genotype *i* will have higher SPVL than individuals infected with genotype *k*, but on average they will.

The means log_10_ SPVL for the viral genotypes, *μ_i_*, are in the range 2.0–7.0, and SPVL phenotypes, *V_j_*, are in the range 0.0–9.0, discretised with step 0.05 and 0.025 respectively. An individual carrying genotype *i*, will have a phenotype *j* with a probability denoted by *f_ij_* which is taken from a normal distribution with mean *μ_i_* and variance *σ_E_* ^2^ (2.2), normalised to sum to one for each genotype *i*.
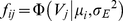
(2.2)


### Prevalence

The prevalence of infections with viral genotype *i*, SPVL phenotype *j*, and age of infection *a* is represented by *Y_ij,a_*(*t*) at time point *t*. The age of infection is the time since the individual was infected. During the course of an infection each host passes through three stages, primary, asymptomatic and disease (AIDS) (P, A and D) as the age of infection *a* increases.

### Duration of infection

Primary and disease stages have equal duration (*D_P_* and *D_D_*) and rate of transmission (*β_P_* and *β_D_*), regardless of SPVL. Duration of and rate of transmission during asymptomatic infection are dependent on SPVL and the relationships were modelled as Hill functions as fitted in Fraser et al. [19], from which the parameter values relating to these functions were also taken (**Table S1**). The mean duration of the asymptomatic stage of infection for a given SPVL *j* is given by:
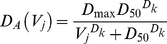
(2.3)The progression from asymptomatic to disease stage is governed by a survival function in **Text S1** equation (5.1), in which *SP_j,a_* is the probability of an individual with SPVL *V_j_* remaining AIDS free at age of infection *a*. This is illustrated in **Figure S1**.

### Rate of transmission

The unadjusted rate of transmission during this stage is given by:
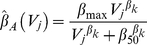
(2.4)Rates of transmission are adjusted for duration and partner change rate, *c*, in order to apply to a serial monogamy model (5.2).

### Force of infection

The rate of transmission, *β_j,a_*, is given in equation (5.3) which incorporates the different stages of infection and the curve for survival during asymptomatic infection. The force of infection for genotype *i* at time *t*, is calculated in equation (2.5) where Δ*t* is the size of the time-step.
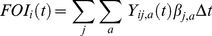
(2.5)


### Mutation

Between generations a between-host mutation step occurs, so the force of infection for genotype *k* seeds a distribution of genotypes. The probability *m_ik_* of an infection with genotype mean *μ_k_* mutating so as to seed a new infection with genotype mean *μ_i_* is taken from a normal distribution with mean *μ_k_* and variance *σ_M_* ^2^ (2.6), normalised to sum to one for each genotype *k*.
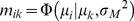
(2.6)Note that this is not mutation in the genetic sense, but rather a measure of the change in the distribution of viral genotypes that occurs over the course of infection within the host.

This model for the change that occurs from one infection to the next, defined by equation (2.6), represents the simplest possible model of the effect of within-host evolution on the distribution of transmitted viruses. More complex models, with directional and host-dependent selection, could feasibly be encoded in more complex mutational matrices.

### New infections in each time-step

The total number of infections for a given genotype in the next time step, *t*+*Δt*, is calculated by the sum of the elementwise product of each *FOI_k_* and the probability that it will mutate into genotype *i*, *m_ik_*. This is scaled according to *X(t)*, the proportion of susceptibles in the population at time *t*, meaning that the genotypes are competing for the available pool of susceptibles. To give the prevalence for each genotype and its SPVL category in the next set of new infections (where *a* = 0), this value is multiplied by the probability of genotype *i* producing SPVL category *j*, *f_ij_*.
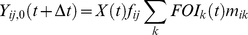
(2.7)


### Update infections

The prevalent infections are updated as in equation (2.8). The term *SP_j,a_* is the function of survival from progression to AIDS, given in equation (5.1). Since AIDS is a stage of determined length, *D_D_*, the function of survival from death at age of infection *a* is given by 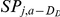, the probability of surviving progression to AIDS at a time *D_D_* years previously.
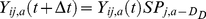
(2.8)


### Update susceptibles

The terms *X_out_*(*t*) and *X_in_*(*t*) refer to new infections and deaths, respectively.
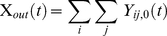
(2.9)

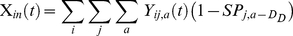
(2.10)These are used to update the susceptible pool, with new infections being removed and individuals who die of AIDS being replaced in the population.
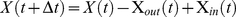
(2.11)


### Calculating *R_0_* for each genotype

The basic reproductive rate, *R_0_*, can be calculated for each genotype, and this can be used to calculate the genotype distribution at equilibrium using the next-generation formalism. The *R_0_* of each genotype is calculated in two steps. Firstly the transmission potential is calculated for an infection with SPVL category *j* by multiplying the rate of transmission in each of the three stages of infection by the length of that stage. The duration of asymptomatic infection *D_A_*(*V_j_*) is the mean of the survival curve.
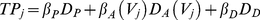
(3.1)Secondly, the basic reproductive rate, *R_0i_*, for each genotype *i*, is then calculated by taking the weighted average transmission potential, *TP_j_*, weighted by the probability that infection with genotype *i* results in infection with SPVL category *j*.
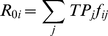
(3.2)


### Solution to equilibrium using next-generation formalism

The R_0_ for each genotype *k* (3.2) and the probability that genotype *k* mutates into genotype *i* (2.6) can be used to calculate the next-generation matrix, *K*.
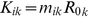
(3.3)The distribution of genotypes at equilibrium is the eigenvector *ε* corresponding to the dominant eigenvalue, *λ*, of *K*.
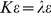
(3.4)The prevalence of SPVL category *j*, *p_j_*, at equilibrium in the population is then calculated as follows.
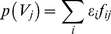
(3.5)This value can then be directly compared with the observed distribution of SPVL.

The likelihood of each run of the model is calculated by comparison with data from a previous study reporting the SPVL of phylogenetically confirmed transmission pairs [6] selected from a cohort in Rakai, Uganda [52], [53]. The likelihood is given by the probability of observing the index SPVL, V_d_, and the recipient's SPVL, *V_r_*. This is calculated using conditional probabilities and is given as follows. The mean log_10_ SPVL of the genotypes infecting the recipient and index case are given by *μ_x_* and *μ_y_*. As these are unknown, all possible combinations of genotypes are considered.
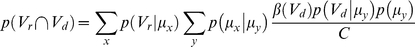
(3.6)in which C is a constant:
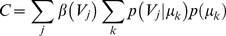
(3.7)and the following have been previously defined in equations (2.2), (2.6) and (3.4):
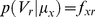
(3.8)

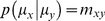
(3.9)

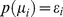
(3.10)The total log likelihood is calculated for each couple *c* in which the direction of transmission is known, and for each couple *u* where the direction is unknown the log likelihood is worked out for each direction and the mean is taken (in this case, *V_m_* and *V_f_* refer to SPVL of males and females, respectively).
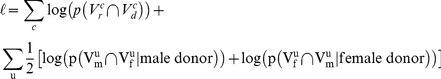
(3.11)


### Calculate heritability

Heritability is the proportion of total variation which is determined by genetic variation in the viral population. It was measured previously by calculating the proportion of the total variance which was explained by carrying genetically similar virus [6]. This can be measured for the modelled distribution in a similar fashion. The non-heritable component is the variance in SPVL in individuals infected by an index partner with a particular SPVL, as a proportion of total variance. This is weighted according to each possible SPVL of the index.
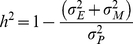
(3.12)

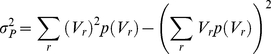
(3.13)


### Likelihood

The likelihood was estimated by calculating the total likelihood, *ℓ_total_*, for each combination of values of *σ_E_* (range 0–1.2, step 0.005) and *σ_M_* (range 0–1.0, step 0.005). Outside of these ranges the likelihood of observing the data is very low, as the variance of the equilibrium distribution becomes vastly higher than is observed. These values were used instead of their squares, *σ_E_* ^2^ and *σ_M_* ^2^, because they are on the same scale as log_10_ SPVL and are therefore directly related to the size of the host effect and of between-host mutation. Furthermore, using *σ_E_* and *σ_M_* gives greater resolution at lower values in the range of interest.

The values of *Y_0_* and *μ_î_* were not included in this analysis as they are not relevant to the equilibrium distribution since they serve only as starting points in the model. All other parameter values were taken from the literature (**Table S1**).

The maximum likelihood combination of these two parameters was estimated and the 95% confidence bounds were identified using a likelihood ratio test (5.4).

### Convergence of SPVL distribution

The next-generation formalism solution described above is sufficient for analysing the equilibrium distribution of SPVL as the end results are identical. However, the model must be run in full to determine the rate at which SPVL evolves in real time.

To run the model in continuous time, the infection is initialised at time *t* = 0 for the starting genotype *î* with mean *μ_î_* and a proportion *Y_0_* of the population are infected. The total number of infected individuals at the start of the epidemic all enter genotype category *î*, and are divided up between all the SPVL categories according to *f_îj_*.
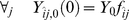
(4.1)All other genotype categories begin at zero, (4.2), as do all ages of infection greater than zero (4.3).
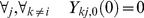
(4.2)

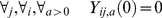
(4.3)The model was run for 500 years in discrete time-steps corresponding to one month for each set of the parameter values.

Parameter values, listed in Table S1, were taken from the literature [19], [54], [55]. Analyses were conducted using C++, MATLAB and R [56]–[58], the latter of which was also used to produce the figures [59].

## Supporting Information

Figure S1 **Possible disease progression outcomes for an infection with log_10_ SPVL of 6.0.** All individuals have the same length of primary and disease stage infection, regardless of SPVL. The survival function is the border between asymptomatic and disease stage infection (“survival” here refers to survival from progression to AIDS, not death). A similar pattern is seen at other SPVL, but with a different survival function.
(TIFF)

Figure S2 **Details of the likelihood surface.** (**a**) For each value of *σ_M_*, the value of *σ_E_* which gives the highest likelihood is marked in orange on the figure, while the yellow region gives the 95% confidence bounds. Similarly, for each value of *σ_E_*, the optimum *σ_M_* value is marked in dark blue, with 95% confidence bounds in light blue. Where the maximum likelihood regions for the two parameters overlap this is marked in green, and the point of maximum likelihood is white. (**b**) Likelihood at the optimum value of *σ_E_* for each value of *σ_M_* i.e. it tracks the likelihood of the orange line. (**c**) Likelihood at the optimum value of *σ_M_* for each value of *σ_E_* i.e. it tracks the likelihood of the dark blue line.
(TIFF)

Figure S3 **Heritability of SPVL measured at equilibrium for each combination of parameters** ***σ_M_*** **and** ***σ_E_*** **.** The black line represents the border of the 95% confidence interval on the maximum likelihood plot, Figure 1.
(TIFF)

Figure S4 **Population variance of SPVL measured at equilibrium for each combination of parameters** ***σ_M_*** **and** ***σ_E_*** **.** The black line represents the border of the 95% confidence interval on the maximum likelihood plot, Figure 1.
(TIFF)

Table S1 **Parameter values.** Where possible these values have been taken from the literature, and a broad range of plausible values are applied to unknown parameters.
(DOC)

Table S2 **The range of values used to construct the latin hypercube sample.** The values for each point in the hypercube were sampled from a uniform distribution within that range.
(DOC)

Table S3 **The maximum likelihood estimates of** ***σ_M_*** **and** ***σ_E_*** **in 1000 bootstraps.** The figures are the proportion of each combination of values of *σ_M_* and *σ_E_* which were the maximum likelihood estimate when a low resolution likelihood surface was calculated with 1000 sets of bootstrapped parameters. These exclude 19 bootstraps in which the optimised parameter values gave a next-generation matrix with mixed signs, rendering the result incalculable.
(DOC)

Table S4 **The combination of parameters with the highest value of** ***σ_M_*** **in 1000 bootstraps.** The figures are the proportion of each combination of values of *σ_M_* and *σ_E_* which formed the highest value of *σ_M_* which was still consistent with the 95% confidence region of the maximum likelihood estimate. Where several values of *σ_E_* were available, the one with the highest likelihood was chosen.
(DOC)

Text S1 **Supporting information containing further details of the methods and results.**
(DOC)
