# Supplementary figures and images for: Transmission Selects for HIV-1 Strains of Intermediate Virulence: A Modelling Approach
Source: PLoS Comput Biol. 2011 Oct 13;7(10):e1002185. doi: 10.1371/journal.pcbi.1002185 (PMC3192807; doi:10.1371/journal.pcbi.1002185)

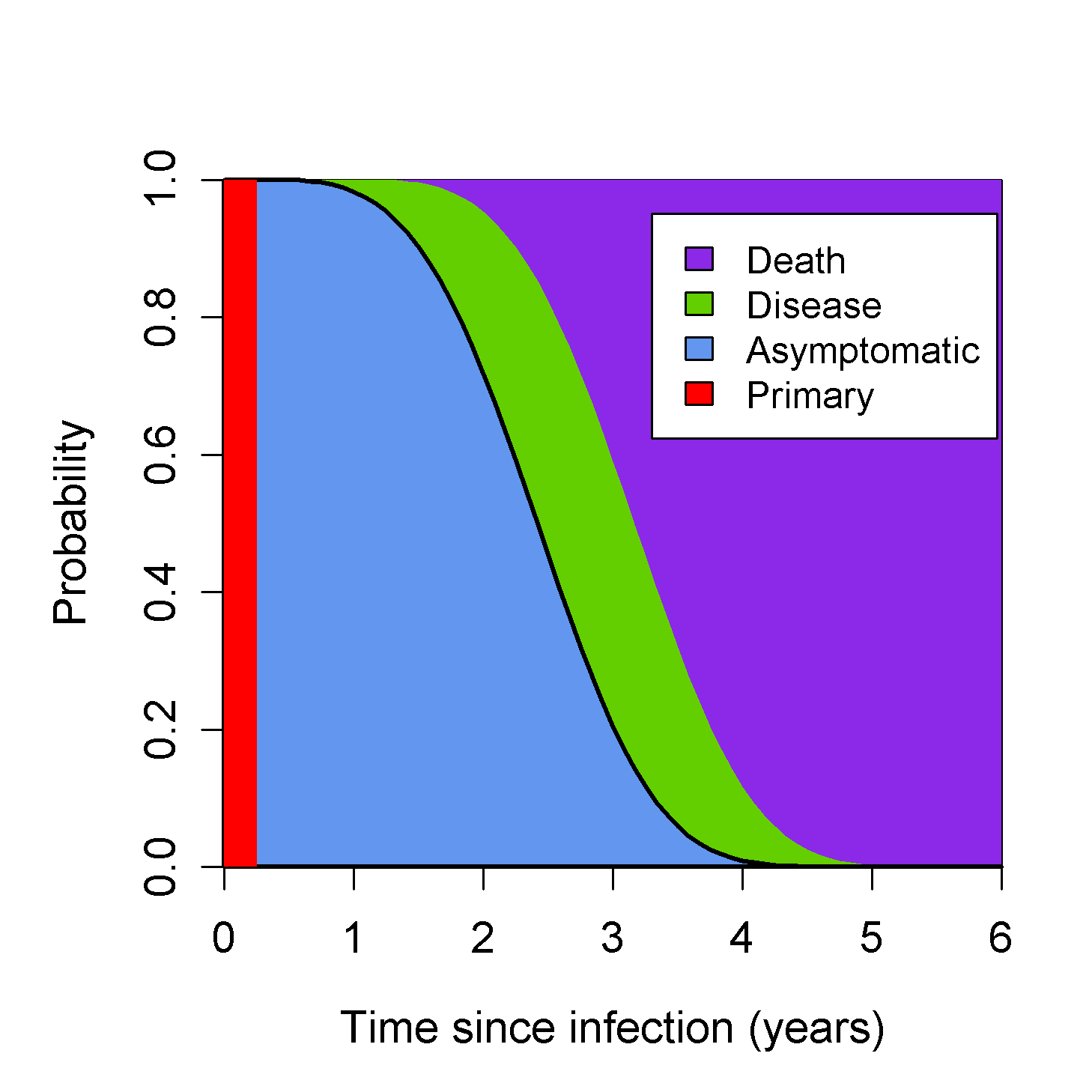

Supplement: Figure S1 — Possible disease progression outcomes for an infection with log10 SPVL of 6.0. All individuals have the same length of primary and disease stage infection, regardless of SPVL. The survival function is the border between asymptomatic and disease stage infection (“survival” here refers to survival from progression to AIDS, not death). A similar pattern is seen at other SPVL, but with a different survival function. (TIFF) [file pcbi.1002185.s001.tiff]

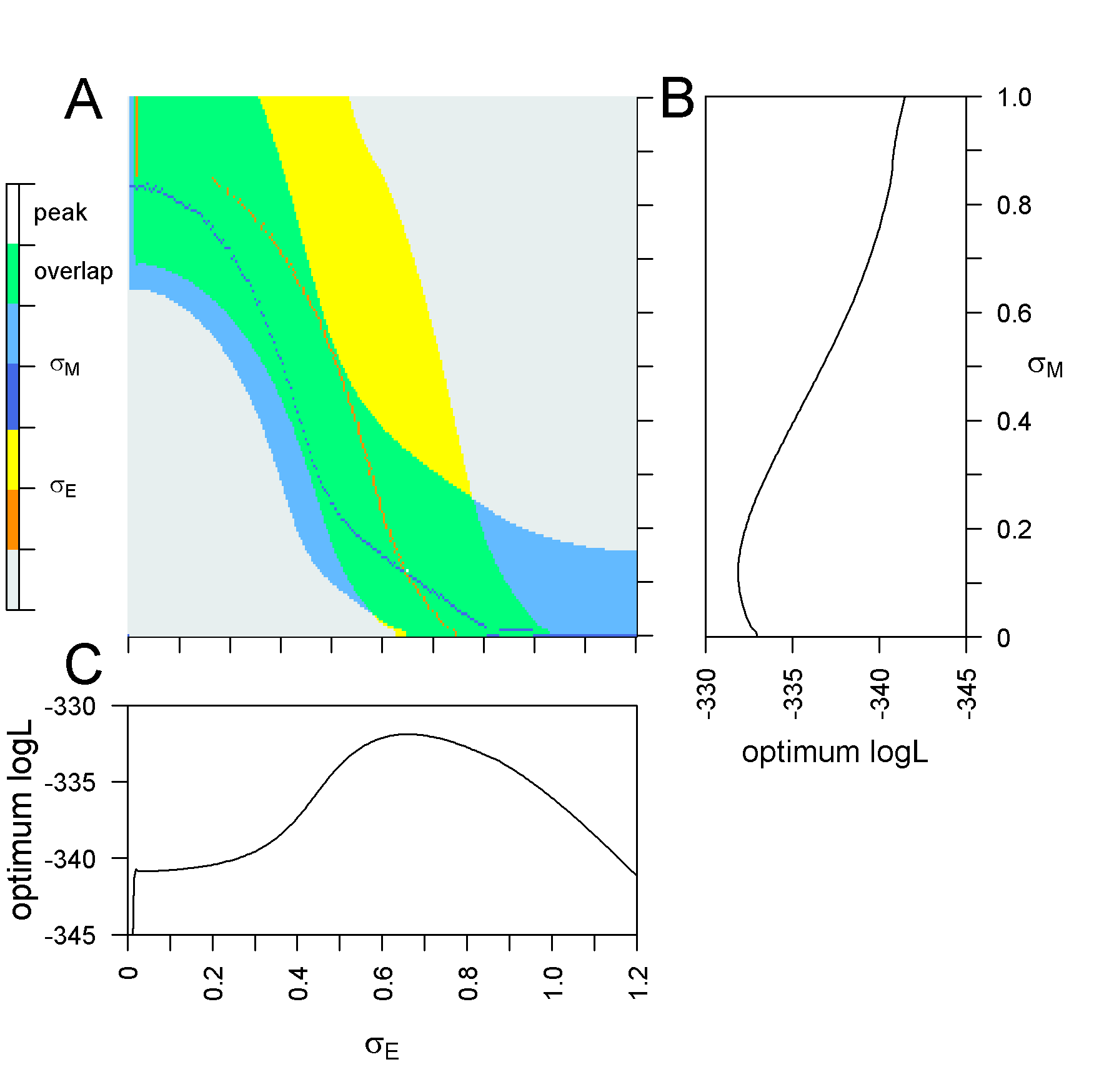

Supplement: Figure S2 — Details of the likelihood surface. (a) For each value of σM, the value of σE which gives the highest likelihood is marked in orange on the figure, while the yellow region gives the 95% confidence bounds. Similarly, for each value of σE, the optimum σM value is marked in dark blue, with 95% confidence bounds in light blue. Where the maximum likelihood regions for the two parameters overlap this is marked in green, and the point of maximum likelihood is white. (b) Likelihood at the optimum value of σE for each value of σM i.e. it tracks the likelihood of the orange line. (c) Likelihood at the optimum value of σM for each value of σE i.e. it tracks the likelihood of the dark blue line. (TIFF) [file pcbi.1002185.s002.tiff]

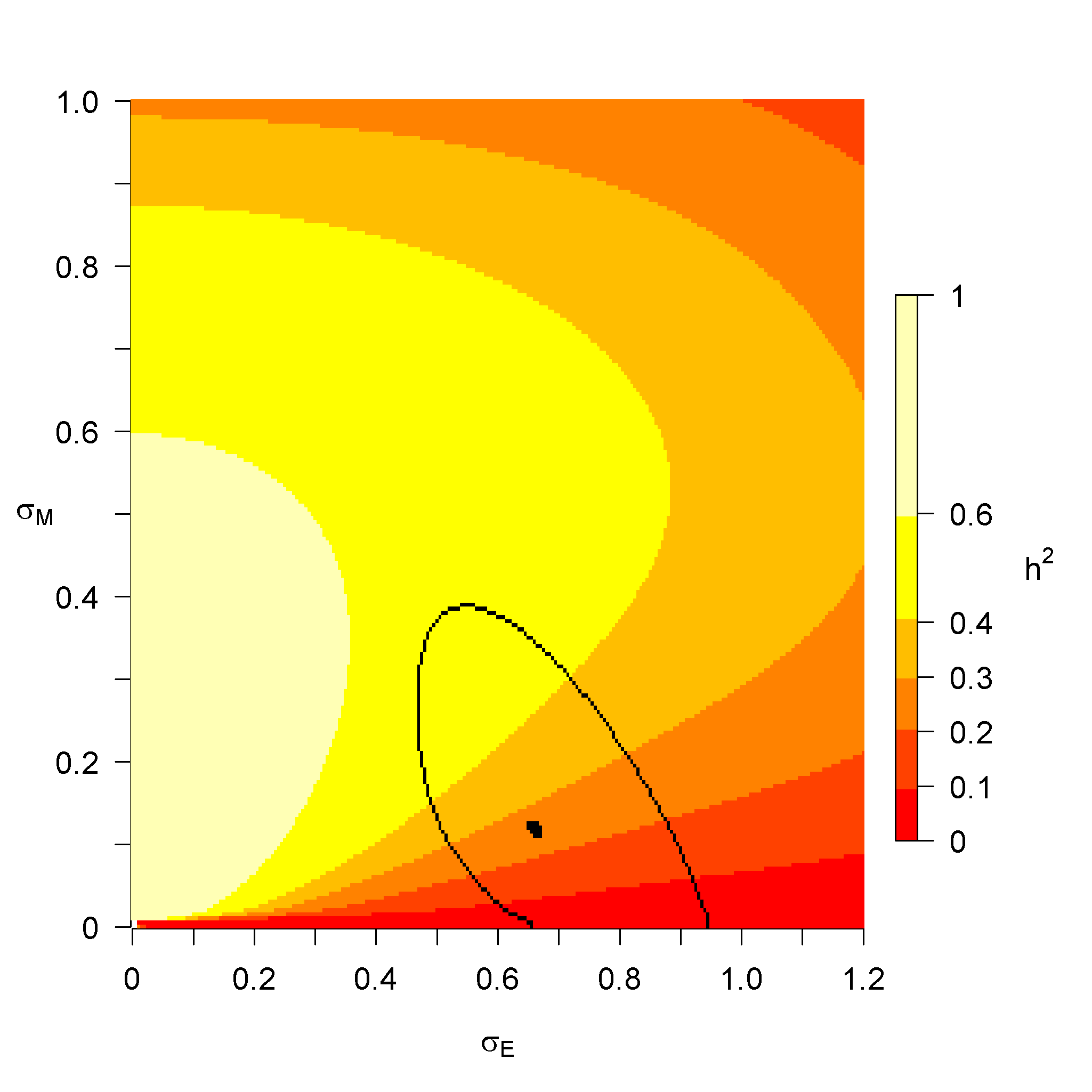

Supplement: Figure S3 — Heritability of SPVL measured at equilibrium for each combination of parameters σM and σE . The black line represents the border of the 95% confidence interval on the maximum likelihood plot, Figure 1. (TIFF) [file pcbi.1002185.s003.tiff]

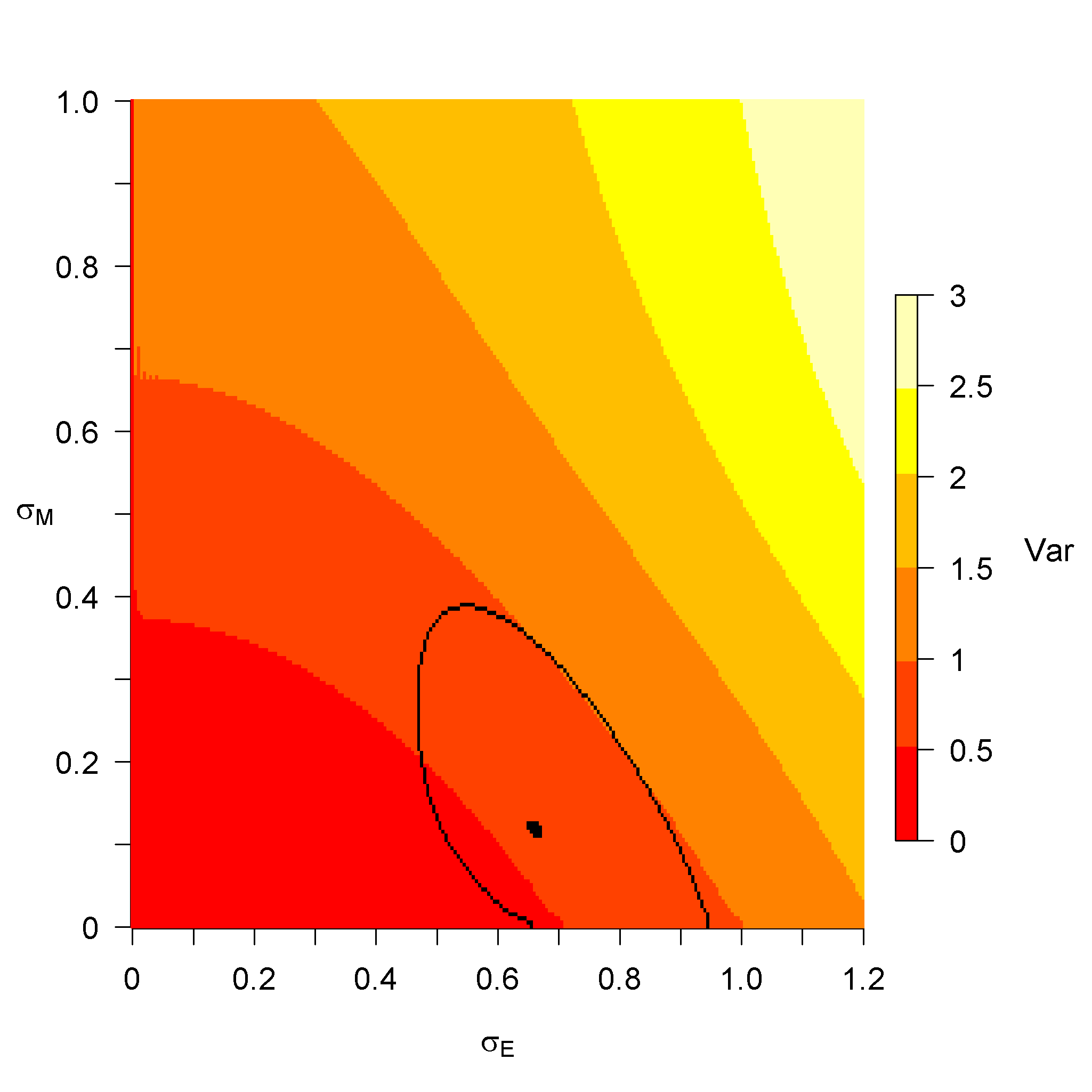

Supplement: Figure S4 — Population variance of SPVL measured at equilibrium for each combination of parameters σM and σE . The black line represents the border of the 95% confidence interval on the maximum likelihood plot, Figure 1. (TIFF) [file pcbi.1002185.s004.tiff]
