# Supplementary material for: Transmission Selects for HIV-1 Strains of Intermediate Virulence: A Modelling Approach
Source: PLoS Comput Biol. 2011 Oct 13;7(10):e1002185. doi: 10.1371/journal.pcbi.1002185 (PMC3192807; doi:10.1371/journal.pcbi.1002185)
Supplement: Table S1 — Parameter values. Where possible these values have been taken from the literature, and a broad range of plausible values are applied to unknown parameters. (DOC) [file pcbi.1002185.s005.doc]

**Supplementary Table 1.** Parameter values.

| Symbol | Description | Value | Reference |
| --- | --- | --- | --- |
| $\hat{\beta}_{P}$ | Unadjusted rate of transmission during primary infection. | 2.76 per year | [54] |
| $\hat{\beta}_{D}$ | Unadjusted rate of transmission during high transmission disease stage. | 0.76 per year | [54] |
| *D_P_* | Duration of primary infection | 0.25 years | [54] |
| *D_D_* | Duration of high transmission disease stage | 0.75 years | [54] |
| *β*_max_ | Maximum rate of transmission during asymptomatic stage | 0.317 per year | [19] |
| *β*_50_ | SPVL at which infectiousness is half maximum | 13,938 copies per ml | [19] |
| *β_k_* | Hill coefficient: steepness of increase in infectiousness as a function of SPVL | 1.02 | [19] |
| *D*_max_ | Maximum duration of asymptomatic stage | 25.4 years | [19] |
| *D*_50_ | SPVL at which duration of asymptomatic infection is half maximum | 3,058 copies per ml | [19] |
| *D_k_* | Hill coefficient: steepness of decrease in duration as a function of SPVL | 0.41 | [19] |
| *ρ* | Shape coefficient for the Weibull survival distribution | 3.46 | [19] |
| *c* | Partner change rate | 1.25 | [55] |
| *σ_E_* | Environmental standard deviation of log_10_ SPVL | 0-1.2 | Unknown |
| *σ_M_* | Mutational standard deviation of log_10_ SPVL | 0-1.0 | Unknown |
|  | Mean log_10_ SPVL of founding genotype | 2.0-7.0 | Unknown |
| *Y_0_* | Proportion infected with founding genotype | 0.001 | Unknown |
