# Supplementary material for: Transmission Selects for HIV-1 Strains of Intermediate Virulence: A Modelling Approach
Source: PLoS Comput Biol. 2011 Oct 13;7(10):e1002185. doi: 10.1371/journal.pcbi.1002185 (PMC3192807; doi:10.1371/journal.pcbi.1002185)
Supplement: Table S2 — The range of values used to construct the latin hypercube sample. The values for each point in the hypercube were sampled from a uniform distribution within that range. (DOC) [file pcbi.1002185.s006.doc]

**Supplementary Table 2.** The range of values used to construct the latin hypercube sample.

| Parameter | Minimum | Maximum |
| --- | --- | --- |
| D_max_ | 1 | 50 |
| D_50_ | 1000 | 10000 |
| D_k_ | 0.01 | 1 |
| ρ | 1 | 5 |
| β_max_ | 0.0001 | 2 |
| β_50_ | 1 | 100000 |
| β_k_ | 0.0001 | 2 |
