# Supplementary material for: Transmission Selects for HIV-1 Strains of Intermediate Virulence: A Modelling Approach
Source: PLoS Comput Biol. 2011 Oct 13;7(10):e1002185. doi: 10.1371/journal.pcbi.1002185 (PMC3192807; doi:10.1371/journal.pcbi.1002185)
Supplement: Table S3 — The maximum likelihood estimates of σM and σE in 1000 bootstraps. The figures are the proportion of each combination of values of σM and σE which were the maximum likelihood estimate when a low resolution likelihood surface was calculated with 1000 sets of bootstrapped parameters. These exclude 19 bootstraps in which the optimised parameter values gave a next-generation matrix with mixed signs, rendering the result incalculable. (DOC) [file pcbi.1002185.s007.doc]

**Supplementary Table 3.** The maximum likelihood estimates of σ_M_ and σ_E_ in 1000 bootstraps.

|  | σ_E_ |  |  |  |  |  |  |  |  |  |  |  |  |
| --- | --- | --- | --- | --- | --- | --- | --- | --- | --- | --- | --- | --- | --- |
| σ_M_ | 0.0 | 0.1 | 0.2 | 0.3 | 0.4 | 0.5 | 0.6 | 0.7 | 0.8 | 0.9 | 1.0 | 1.1 | 1.2 |
| 1.0 | 0 | 0.005 | 0.052 | 0.034 | 0.010 | 0.004 | 0.001 | 0 | 0 | 0 | 0 | 0 | 0 |
| 0.9 | 0 | 0 | 0 | 0 | 0 | 0 | 0 | 0 | 0 | 0 | 0 | 0 | 0 |
| 0.8 | 0 | 0 | 0 | 0 | 0 | 0 | 0 | 0 | 0 | 0 | 0 | 0 | 0 |
| 0.7 | 0 | 0 | 0 | 0 | 0 | 0 | 0 | 0 | 0 | 0 | 0 | 0 | 0 |
| 0.6 | 0 | 0 | 0 | 0 | 0 | 0 | 0 | 0 | 0 | 0 | 0 | 0 | 0 |
| 0.5 | 0 | 0 | 0 | 0 | 0 | 0 | 0.007 | 0.003 | 0 | 0 | 0 | 0 | 0 |
| 0.4 | 0 | 0 | 0 | 0 | 0 | 0 | 0.020 | 0.047 | 0 | 0 | 0 | 0 | 0 |
| 0.3 | 0 | 0 | 0 | 0 | 0 | 0 | 0 | 0.282 | 0.007 | 0 | 0 | 0 | 0 |
| 0.2 | 0 | 0 | 0 | 0 | 0 | 0 | 0 | 0.462 | 0 | 0 | 0 | 0 | 0 |
| 0.1 | 0 | 0 | 0 | 0 | 0 | 0 | 0 | 0.065 | 0 | 0 | 0 | 0 | 0 |
| 0.0 | 0 | 0 | 0 | 0 | 0 | 0 | 0 | 0 | 0 | 0 | 0 | 0 | 0 |
