# Supplementary material for: Transmission Selects for HIV-1 Strains of Intermediate Virulence: A Modelling Approach
Source: PLoS Comput Biol. 2011 Oct 13;7(10):e1002185. doi: 10.1371/journal.pcbi.1002185 (PMC3192807; doi:10.1371/journal.pcbi.1002185)
Supplement: Table S4 — The combination of parameters with the highest value of σM in 1000 bootstraps. The figures are the proportion of each combination of values of σM and σE which formed the highest value of σM which was still consistent with the 95% confidence region of the maximum likelihood estimate. Where several values of σE were available, the one with the highest likelihood was chosen. (DOC) [file pcbi.1002185.s008.doc]

**Supplementary Table 4.** The combination of parameters with the highest value of σ_M_ in 1000 bootstraps.

|  | σ_E_ |  |  |  |  |  |  |  |  |  |  |  |  |
| --- | --- | --- | --- | --- | --- | --- | --- | --- | --- | --- | --- | --- | --- |
| σ_M_ | 0.0 | 0.1 | 0.2 | 0.3 | 0.4 | 0.5 | 0.6 | 0.7 | 0.8 | 0.9 | 1.0 | 1.1 | 1.2 |
| 1.0 | 0 | 0.200 | 0.244 | 0.060 | 0.010 | 0.004 | 0.001 | 0 | 0 | 0 | 0 | 0 | 0 |
| 0.9 | 0 | 0 | 0 | 0.036 | 0 | 0 | 0 | 0 | 0 | 0 | 0 | 0 | 0 |
| 0.8 | 0 | 0 | 0 | 0 | 0.053 | 0 | 0 | 0 | 0 | 0 | 0 | 0 | 0 |
| 0.7 | 0 | 0 | 0 | 0 | 0 | 0.112 | 0 | 0 | 0 | 0 | 0 | 0 | 0 |
| 0.6 | 0 | 0 | 0 | 0 | 0 | 0.162 | 0 | 0 | 0 | 0 | 0 | 0 | 0 |
| 0.5 | 0 | 0 | 0 | 0 | 0 | 0 | 0.082 | 0 | 0 | 0 | 0 | 0 | 0 |
| 0.4 | 0 | 0 | 0 | 0 | 0 | 0 | 0.035 | 0 | 0 | 0 | 0 | 0 | 0 |
| 0.3 | 0 | 0 | 0 | 0 | 0 | 0 | 0.002 | 0 | 0 | 0 | 0 | 0 | 0 |
| 0.2 | 0 | 0 | 0 | 0 | 0 | 0 | 0 | 0 | 0 | 0 | 0 | 0 | 0 |
| 0.1 | 0 | 0 | 0 | 0 | 0 | 0 | 0 | 0 | 0 | 0 | 0 | 0 | 0 |
| 0.0 | 0 | 0 | 0 | 0 | 0 | 0 | 0 | 0 | 0 | 0 | 0 | 0 | 0 |
