# Supplementary material for: Transmission Selects for HIV-1 Strains of Intermediate Virulence: A Modelling Approach
Source: PLoS Comput Biol. 2011 Oct 13;7(10):e1002185. doi: 10.1371/journal.pcbi.1002185 (PMC3192807; doi:10.1371/journal.pcbi.1002185)
Supplement: Text S1 — Supporting information containing further details of the methods and results. (DOC) [file pcbi.1002185.s009.doc]

# Text S1

## Survival function

The probability, *SPj,a*, of an individual with viral load *Vj* remaining AIDS-free at age of infection *a* is given by a survival function [19] where *DA*(*Vj*) is the average duration of asymptomatic infection from equation (2.3).

$SP_{j,a}=\left\{\begin{matrix} 1 0\leq a<D_{P} \\ e^{-\left(\frac{\left(a-D_{P}\right)\exp\left(\ln\Gamma\left(1+\frac{1}{\rho}\right)\right)}{D\left(V_{j}\right)}\right)^{\rho}} D_{P}\leq a \end{matrix}\right.$ (5.1)

## Adjustment for serial monogamy

Each of the rates of transmission in all three stages, *S*, are adjusted in equation (5.2) to approximate a serial monogamy model with a partner change rate *c* and the duration of the stage, *DS*, according to the method in Hollingsworth et al. [54].

$\beta_{Sj}=\frac{\hat{\beta}_{Sj}c}{\hat{\beta}_{Sj}+c+\frac{1}{D_{Sj}}}$ (5.2)

## Disease stages and rate of transmission

The rate of transmission for SPVL category *j* and age of infection *a* is given in equation (5.3). It is equivalent to *βP* for the duration of primary infection DP but thereafter a proportion of individuals of SPVL category *j* is in asymptomatic infection according to the survival probability *SP­j,a*. The proportion that enter the disease stage remain in it for a fixed period of time (*DD*) before death, and the rate of transmission at that age of infection reflects the proportions of individuals in asymptomatic stage, disease stage, and death.

(5.3)

This is illustrated for an example SPVL in **Figure S1**.

## Maximum likelihood confidence intervals

The confidence intervals in the likelihood plot were identified using a likelihood ratio test (5.4). Here $\chi_{\delta}^{2}\left(\alpha\right)$is the point on the chi-square distribution with *δ* degrees of freedom which encompasses a proportion (1-*α*) of the area under the curve (1.39, 5.99 and 13.82 when *α*=0.50, 0.05 and 0.001 respectively). is the maximum log likelihood value, and is the log likelihood for combination of parameters *σM* and *σE*. If the inequality is satisfied, then the values *σM* and *σE* produce a log likelihood not significantly different from the maximum likelihood at a 100(1-*α*)% confidence.

(5.4)

## Expected distribution of recipient and index SPVL

The probability of a given SPVL, *p*(*Vr*), as given in equation (3.5) is equivalent to the probability of observing a given SPVL in a recipient. The probability of observing a given SPVL in an index case is conditional on the index partner’s rate of transmission, so the value is weighted accordingly:

$p\left(V_{d}\left|T\right.\right)=\frac{\beta\left(V_{d}\right)p\left(V_{d}\right)}{\sum_{j} \beta\left(V_{j}\right)p\left(V_{j}\right)}$ (5.5)

## Bootstrap

The bootstrap was performed to refit the relationship between the data and the parameters, as reported in Fraser et al. [19]. The data had its source in papers published on studies from Amsterdam [12] and Zambia [18]. The relationship between SPVL and duration of infection, and between SPVL and rate of transmission, were each fitted to a Hill function as this is sufficiently general and biologically relevant.

To measure the fit of the model for the duration of infection, the likelihood was calculated for each individual, *s*, and summed as follows. Weibull(*x, α, β*) is the probability density function at *x* for the Weibull distribution with parameters *α* and *β*. Γ(*x*) is the gamma function at *x*, and Γc(*x, α, β*) is the cumulative at *x* of the gamma distribution with parameters *α* and *β*. The value *Ds* is the time that the individual is followed up, regardless of outcome, and *D*(*Vs*) is the expected time to progression given the individual’s SPVL *Vs* which is calculated from equation (2.3).

(6.1)

The likelihood for the transmission data was calculated similarly from the probability of an individual transmitting over the length of the observed period. The rate of transmission *β*(*Vs*) was calculated for each individual, *s*, from their SPVL as in equation (2.4). The probability of transmission was given as follows, where *T*=1.833, the length of the study period.

(6.2)

The bootstrap was performed by sampling with replacement the relevant dataset, and then fitting the parameters to this data using the *optim* command in R [56] which adjusts the parameters to maximise the likelihood. The starting point for the fit was chosen by latin hypercube sampling in the *tgp* package [57,58] to cover the available parameter space. Initial values were selected at random uniformly between the values in **Table S2**.

The bootstrap was repeated 1000 times for each relationship, and then a set of duration parameters was randomly assigned to a set of transmission parameters. These were then used as the parameters in the model. For each of these sets of parameters the maximum likelihood estimates for *σM* and *σE* were calculated(**Table S3**) The step size of *σM* and *σE*­ was 0.1 instead of 0.005 to reduce computation time. The point with the highest value of *σM* which was still of significant likelihood was also identified (**Table S4**).

## Discretisation

The mean log10 SPVL of the viral genotypes and the categories of SPVL they produce were discretised in steps of 0.1 log10. Furthermore time was discretised into steps of one month. These step sizes were chosen for their convenience and were believed to give an appropriate level of resolution while keeping the computation time manageable. Reducing the discretisation step size had a minimal effect on the results (data not shown).

## Starting prevalence

Finally, we also explored the effect of the proportion infected at the start, *Y0*, and found that this had a negligible effect on the rate of mean convergence when the founding virulence was high, and an insubstantial effect when founding virulence was low (data not shown). The effect of discretisation was tested by reducing the SPVL phenotype and viral genotype step size to 0.01, and the time-step was reduced to 0.01 years. Upon repeating the analysis, there was a negligible difference in the results (data not shown).
